# The Evolution of the *DLK1-DIO3* Imprinted Domain in Mammals

**DOI:** 10.1371/journal.pbio.0060135

**Published:** 2008-06-03

**Authors:** Carol A Edwards, Andrew J Mungall, Lucy Matthews, Edward Ryder, Dionne J Gray, Andrew J Pask, Geoffrey Shaw, Jennifer A.M Graves, Jane Rogers, the SAVOIR consortium, Ian Dunham, Marilyn B Renfree, Anne C Ferguson-Smith

**Affiliations:** 1 Department of Physiology, Development, and Neuroscience, University of Cambridge, Cambridge, United Kingdom; 2 Wellcome Trust Sanger Institute, Wellcome Trust Genome Campus, Hinxton, Cambridge, United Kingdom; 3 Department of Genetics, University of Cambridge, Cambridge, United Kingdom; 4 Department of Zoology, University of Melbourne, Victoria, Australia; 5 Research School of Biological Sciences, The Australian National University, Canberra, Australia; Oxford University, United Kingdom

## Abstract

A comprehensive, domain-wide comparative analysis of genomic imprinting between mammals that imprint and those that do not can provide valuable information about how and why imprinting evolved. The imprinting status, DNA methylation, and genomic landscape of the *Dlk1-Dio3* cluster were determined in eutherian, metatherian, and prototherian mammals including tammar wallaby and platypus. Imprinting across the whole domain evolved after the divergence of eutherian from marsupial mammals and in eutherians is under strong purifying selection. The marsupial locus at 1.6 megabases, is double that of eutherians due to the accumulation of LINE repeats. Comparative sequence analysis of the domain in seven vertebrates determined evolutionary conserved regions common to particular sub-groups and to all vertebrates. The emergence of *Dlk1-Dio3* imprinting in eutherians has occurred on the maternally inherited chromosome and is associated with region-specific resistance to expansion by repetitive elements and the local introduction of noncoding transcripts including microRNAs and C/D small nucleolar RNAs. A recent mammal-specific retrotransposition event led to the formation of a completely new gene only in the eutherian domain, which may have driven imprinting at the cluster.

## Author Summary

Mammals have two copies of each gene in their somatic cells, and most of these gene pairs are regulated and expressed simultaneously. A fraction of mammalian genes, however, is subject to imprinting—a chemical modification that marks a gene according to its parental origin, so that one parent's copy is expressed while the other parent's copy is silenced. How and why this process evolved is the subject of much speculation. Here we have shown that all the genes in one genomic region, *Dlk1-Dio3*, which are imprinted in placental mammals such as mouse and human, are not imprinted in marsupial (wallaby) or monotreme (platypus) mammals. This is in contrast to a small number of other imprinted genes that are imprinted in marsupials and other therian mammals and indicates that imprinting arose at each genomic domain at different stages of mammalian evolution. We have compared the sequence of the *Dlk1-Dio3* region between seven vertebrate species and identified sequences that are differentially represented in mammals that imprint compared to those that do not. Our data indicate that once imprinted gene regulation is acquired in a domain, it becomes evolutionarily constrained to remain unchanged.

## Abstract 3

A comparative analysis of genomic imprinting between mammals that imprint and those that don't has provided insights into how and why imprinting evolved.

## Introduction

Genomic imprinting is a process that causes genes to be expressed according to their parental origin and is evident in plants and mammals. Many imprinted genes are located in clusters regulated by a single imprinting control element, whose function across the whole imprinted domain depends on DNA methylation acquired differentially in the male and the female germlines [1]. It is not known how or why mammalian imprinting evolved; however, its emergence is associated with the evolution of a placenta [2,3], and the correct dosage of imprinted genes is important in prenatal growth, postnatal metabolism [4], and neurodevelopment [5]. Where tested, the majority of imprinted genes are expressed and imprinted, sometimes specifically, in the placenta [6], suggesting that even distantly related placental mammals such as metatherians (marsupials) will have imprinting, while oviparous mammals, the prototherians (monotremes), will not. Assessment of the imprinting status of a few individual mammalian imprinted genes is consistent with these data. The orthologues of four genes imprinted in mouse and human are clearly imprinted in marsupials [7–10], and no evidence of imprinting has been found in monotremes, although only three genes have been tested to date [8,11,12].

**Figure 1:**
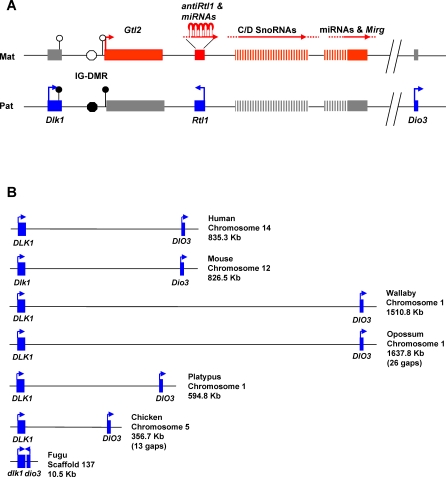
*Dlk1-Dio3* in Vertebrates (A) Schematic representation of the *Dlk1-Dio3* domain in mouse showing genes expressed from the paternal chromosome (blue) and noncoding RNAs (red) expressed from the maternally inherited chromosome. The imprinting control region for the domain is the paternally methylated IG-DMR (circle). Also shown are differentially methylated regions in exon 5 of *Dlk1* and the promoter region of *Gtl2*. Filled circles, methylated; open circlesn unmethylated. Not drawn to scale. (B) The relative positions of *DLK1* and *DIO3* in vertebrates. The domain sizes were calculated from the start codon of *DLK1* to the stop codon of *DIO3*. (Genome builds were human March 2006, mouse February 2006, opossum January 2006, chicken May 2006, and fugu October 2004).

The *Dlk1-Dio3* imprinted domain in eutherian mammals contains the protein-coding genes *Delta-like homologue 1* (*Dlk1*), *Retrotransposon-like gene 1* (*Rtl1/Mart1*), and the type 3 deiodinase (*Dio3*) expressed from the paternally inherited chromosome, and multiple long and short non–protein coding RNAs including microRNAs (miRNAs) and C/D small nucleolar RNA (snoRNA) genes expressed solely from the maternally inherited chromosome (Figure 1A). Seven imprinted miRNAs are located within *anti-Rtl1*, and over forty are located further downstream including within the miRNA-containing gene *Mirg* (Figure 1A). All of the genes in the domain are developmentally regulated and expressed in a range of embryonic and extraembryonic cells types with postnatal expression being found predominantly in the brain [13–15]^.^ In mouse, imprinting is regulated by an intergenic differentially methylated region (IG-DMR), located 75 kb downstream of *Dlk1*, that becomes methylated during spermatogenesis but remains unmethylated in the maternal germline [16,17]. When a targeted deletion of the IG-DMR is inherited maternally, an epigenetic switch occurs causing the maternally inherited chromosome to behave like the paternally inherited chromosome; no effect is seen when the deletion is paternally inherited. The IG-DMR is also differentially methylated in human [17], and recently identified patients with deletions and epimutations in the *DLK1-DIO3* region indicate that this element likely acts as the imprinting control element in human [18]. Tight linkage and strong conservation of *Dlk1* and *Dio3* is maintained in all vertebrates. The two genes are located 10.5 kb apart in Takifugu rubripes, approximately 370 kb apart in chicken, and 830 kb in human and mouse (Figure 1B).

## Results

To determine the sequence and organization of the region in marsupial and monotreme mammals, we cloned and sequenced the region between *DLK1* and *DIO3* in the platypus, Ornithorhynchus anatinus, and the tammar wallaby, Macropus eugenii. Bacterial artificial chromosome (BAC) clones containing the orthologous *DLK1* and *DIO3* genes were identified [19]. Thirteen overlapping wallaby BACs and seven overlapping platypus BACs were isolated from genomic libraries, then initially characterized using a parallel landmark content mapping and fingerprinting strategy [20], and sequenced (Figure S1 and Table S1). This genomic sequence represents complete coverage of the domain in both species and was generated independently of the whole-genome sequencing projects for these organisms. The wallaby sequence is 1,510.8 kb and slightly smaller than that of the South American marsupial Monodelphis domestica (1,637.8 kb plus 26 gaps). The marsupial region is therefore approximately twice as long as its eutherian orthologue (Figure 1B). The region in platypus is 594.8 kb, which is 28% smaller than in mouse.

**Figure 2:**
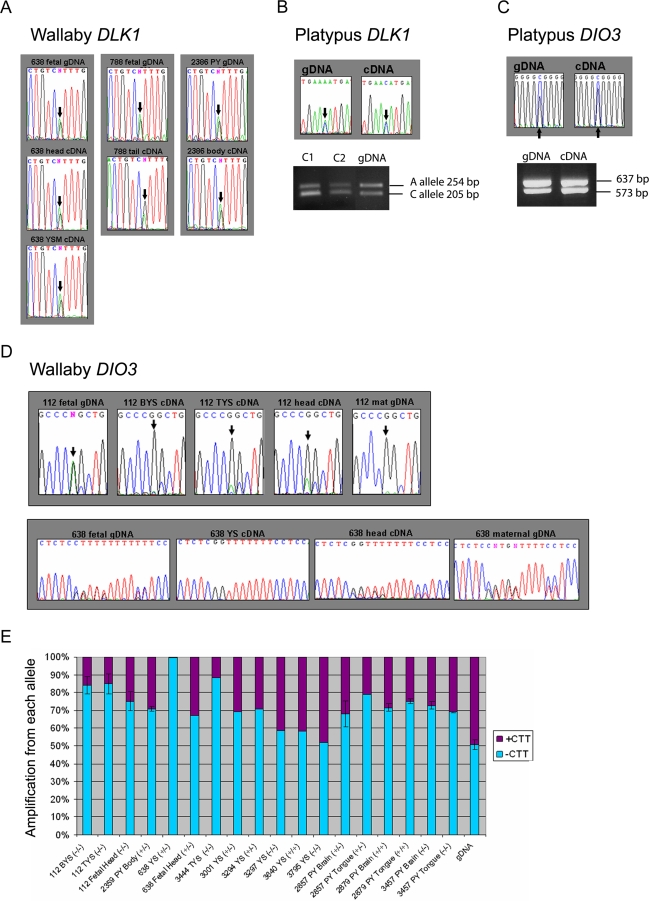
Biallelic Expression of *DLK1* and *DIO3* in Wallaby and Platypus (A) *DLK1* is biallelically expressed in tammar wallaby. The imprinting status of wallaby *DLK1* was determined by analyzing cDNAs shown here from three individuals (638, 788, and 2386) heterozygous for a G/A single nucleotide polymorphism (SNP) in exon 4 at 374 bp from translational start. Biallelic expression was observed in yolk sac placenta (YSM), fetal head, fetal tail, and pouch young (PY) body. Results were confirmed with three further SNPs in the 5′ UTR (Figure S2). (B) *DLK1* is biallelically expressed in platypus. An A/C SNP was identified in the 3′ UTR of the platypus *DLK1* gene 1,323 bp from the translational start. Sequence analysis of cDNA generated from an informative platypus primary fibroblast cell line demonstrated biallelic expression. The C allele of the SNP introduces an *Nla*III into the region. RFLP analysis confirms biallelic expression of platypus *DLK1*. (C) *DIO3* is biallelically expressed in the platypus. Two polymorphisms in platypus *DIO3* were identified in two different primary fibroblast cell lines—a G/C SNP and a 64 bp indel. RT-PCR analysis demonstrates biallelic expression. (D) Two polymorphisms were identified in wallaby DIO3, a CTT indel and a G/A SNP. Preferential expression was observed from the –CTT/G allele, which was particularly evident in yolk sac placenta samples. (E) Quantitative RT-PCR was used to assess the expression from each *DIO3* allele in 12 different heterozygous individuals compared with a standard curve of two gDNA mixed at different ratios. Genomic DNA from all individuals was also tested and compared to the standard curve. Where more than one cDNA was analysed the data were combined and ± standard error are shown. All tissues tested displayed biased expression of the –CTT allele regardless of its parent of origin. BYS, bilaminar yolk sac; TYS, trilaminar yolk sac; YS, yolk sac; and mat, maternal gDNA. The maternal genotype for each individual is are shown in parentheses.

For both wallaby and platypus, *DLK1* and *DIO3* genes were identified, cDNAs characterized, and the genes subjected to imprinting analysis (Figure 2 and Figures S2 and S3). For wallaby, fetal tissues, yolk sac placenta, and pouch young samples were dissected. Platypus fetal material is unavailable, so the analysis was conducted on primary adult skin fibroblasts cultured from two male and one female platypus; therefore the analysis in that species is limited. Several single nucleotide polymorphisms (SNP) (Figure 2A) were identified for *DLK1* from wallaby tissues that included one sample (2386) from a homozygous mother allowing allele-specific activity to be determined. Both maternally and paternally inherited alleles of *DLK1* were expressed in all wallaby fetal, extraembryonic, and pouch young tissues analysed (Figure 2A and Figure S2C). Similar SNP and restriction fragment length polymorphism analysis showed biallelic expression of *DLK1* in platypus (Figure 2B).

Two polymorphisms were identified in wallaby *DIO3*, a G/A SNP at nucleotide 94 in the coding region of the gene and a CTT insertion/deletion (indel) at nucleotide 1,187 in the 3′ untranslated region (UTR) (Figure 2A and Figure S3). Both polymorphisms were present in nine animals, suggesting co-segregation of the variant alleles. Direct sequencing of cDNAs amplified across both polymorphisms indicated there was preferential expression from the G/-CTT allele (Figure 2D). Quantitative real-time, reverse-transcriptase PCR (RT-PCR) proved that wallaby *DIO3* was expressed from both parental chromosomes. However, an allelic bias towards the -CTT allele was observed in all samples tested regardless of parental origin (Figure 2E). Expression analysis of two polymorphisms in platypus *DIO3* confirmed biallelic expression in this species (Figure 2C).

**Figure 3:**
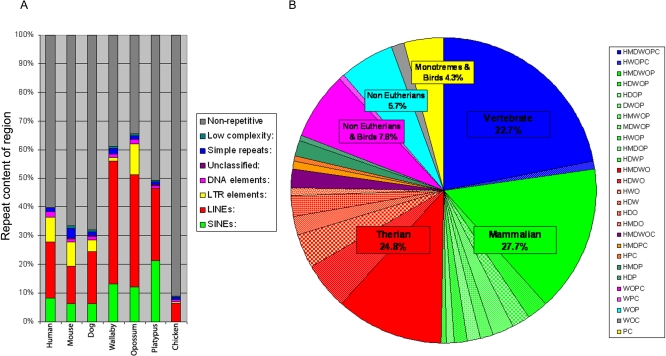
The Genome Landscape and ECRs (A) Repeat content of the *DLK1-DIO3* region in seven vertebrates. The region in both marsupials contains greater than 60% repeats, most of which are LINE1s. The platypus region contains approximately 50% repeats—this is a higher proportion than identified in the eutherian domain despite the region being 28% smaller in platypus. The platypus domain is depleted in LTR repeats. (B) Distribution of the 141 ECRs identified. ECR groups are arranged according to the sub-classes of vertebrates they are identified in. Vertebrate: identified in at least one eutherian, one marsupial, platypus, and chicken. Mammalian: identified in at least one eutherian, one marsupial, and platypus. Therian: identified in three therians including one eutherian and one marsupial. H, human; M, mouse; D, dog; W, wallaby; O, opossum; P, platypus; C, chicken.

Comparative sequence analysis of the *Dlk1-Dio3* genomic landscape between eutherian and other noneutherian mammals can identify the dynamic changes that are associated with and have the potential to contribute to imprinting. Figure 3A and Table 1 show the relative GC and repeat sequence content of the region in seven genomes; three eutherian species (human, mouse, dog), two marsupials (opossum and tammar wallaby), one monotreme (platypus), and one bird (chicken). The eutherian GC content, %CpG and number of CpG islands was significantly higher than the genome average (*p* < 0.01 using Chi-squared test) in contrast to marsupial and monotreme mammals, and chicken, that all lack imprinting at this domain. Repeat content was analysed using the most recent previously unreleased platypus repeat database (kindly provided by R Hubley, Repeatmasker). Eutherian LINE content is consistent with the genome-wide average; however, there is a paucity of LINEs in the region between *Dlk1* and *Mirg* (miRNA-containing gene) in the eutherians (Table 1). The majority of repeats identified in the *DLK-DIO3* domain in the marsupials are LINE1 repeats. This is consistent with the high number of LINEs identified in the opossum genome and suggests that expansion in the *DLK1-DIO3* region, as in the marsupial genome as a whole, is due to LINE1 insertion. The opossum region has a slightly larger proportion of SINEs than expected from the genome average. The SINE content is also greater in the tammar wallaby, although the whole-genome sequence for this species is not currently available for comparison. The relative repeat content in platypus is greater than eutherians despite the region being smaller in this species (Figure 3A). The majority of repeats in the platypus *DLK1-DIO3* region are SINEs and the more ancient LINE2s. Interestingly; there is a notable absence of long terminal repeat (LTR) elements at this locus in platypus (Figure 3A). The chicken region is devoid of any SINE elements which is consistent with the whole genome analysis of this species. Hence platypus and marsupials have greater SINE content in the domain than do the eutherian mammals with imprinting. This is consistent with the SINE depletion previously reported when comparing imprinted with nonimprinted domains in mouse and human [21,22]. Together, these findings indicate that selection against SINE repeats is an evolutionary feature of imprinted domains (see Discussion).

**Table 1:**
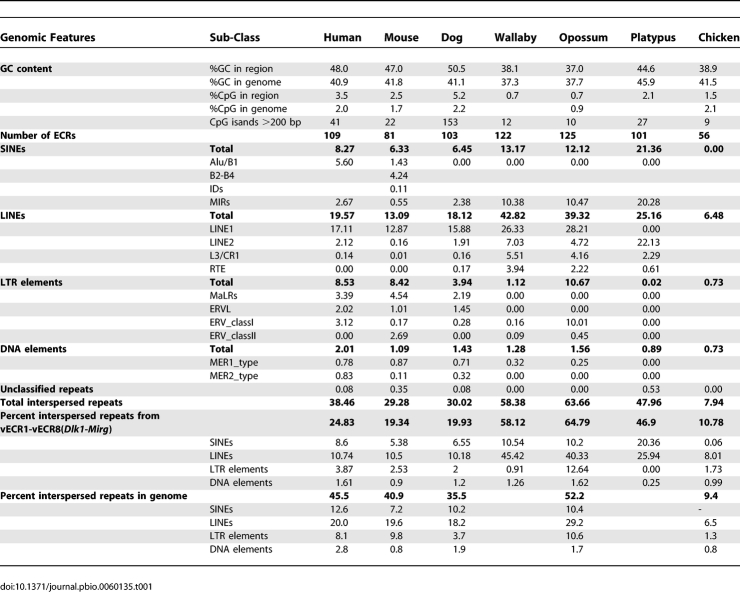
GC and Repeat Content of the *Dlk1-Dio3* Region in Vertebrates

Detailed comparative sequence analysis was conducted between the *Dlk1-Dio3* domain in the seven vertebrates. Using a threshold of 55% nucleotide sequence identity over 80 bp, which recognizes the *Dlk1* exons in all seven sequences, 141 evolutionary conserved regions (ECRs) were identified across sub-groups representing eutherians, marsupials, platypus, and chicken (Table S2). Of the 141 ECRs found, 22.7% (31) were common to all seven vertebrates, 15.6% (22) were common to all mammals, and another 16 were found in all therian mammals. Six were found only in platypus and chicken. Figure 3B illustrates the number of ECRs arranged according to the sub-classes of vertebrates in which they are identified. In mammals, 27.7% were identified in at least one eutherian, one marsupial and platypus, whereas 24.8% were found in at least one species representing each therian infraclass. Although the greatest number of ECRs is found within the mammalian species, the more ancestral ECRs (the 31 found in all species studied) are on average larger, having a mean length of 494 bp compared with the mean length of all ECRs at 340 bp and suggesting greater functional constraint.

**Figure 4:**
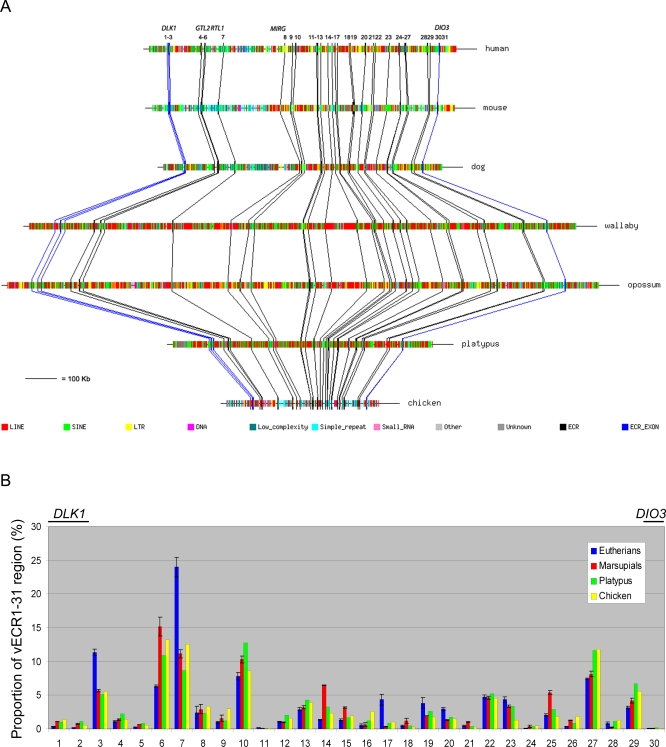
Comparative Analysis of Inter-ECR Zones in the *DLK1-DIO3* Domain (A) ECRs identified in all seven species are shown as blue (*Dlk1* and *Dio3* exons) or black lines. The ECRs are linked to their orthologues in the neighbouring species in order to illustrate the repeat content and relative expansions/contraction within each sequence. (B) The length of each inter-ECR zones from vECR1 (*DLK1* exon 3) to vECR31 (*DIO3*) as a proportion of the length of the domain in eutherians, marsupials, platypus, and chicken. Zone 1 = vECR1–vECR2, zone 2 = vECR2–vECR3, etc. Mean ± standard error for the three eutherians and the two marsupials are shown.

We used the 31 ECRs found in all vertebrates to align the *Dlk1-Dio3* domain and subdivide it into 30 inter-ECR zones for further comparative analysis (Figure 4A). Exons of *Dlk1* and *Dio3* are represented by vertebrate ECRs 1–3 and 30–31, respectively. The intergenic distribution of the ECRs is not uniform throughout the domain with two-thirds being located in the 3′ half of the domain. One of the ECRs, approximately 3 kb upstream of *DIO3,* contains a highly conserved putative CTCF binding site in all therian species.

The amount of sequence in each of the 30 inter-ECR zones relative to the overall size of the domain was quantified for each vertebrate (Figure 4B). This provides a measure of the overall expansions/contractions between species. The regional changes between marsupial, monotreme, and eutherian mammals across the domain are not uniform. The most striking differences between the mammals lie in zone 3 (between vECR3 and vECR4), zone 6, and zone 7. Zone 3 which is located between the last exon of *Dlk1* and the conserved intron 5 region of *Gtl2*, is expanded in eutherians (Figure 4B). This expansion does not appear to be caused by LINEs, because LINE1 and LINE2 repeats are equivalently represented in eutherians and marsupials (Figure S4). As shown, this zone contains a higher proportion of SINE elements than reported for the whole genome and compared with the entire domain. However, the increased SINE content does not explain the expansion of zone 3, which is due to the acquisition of unique sequence, including the imprinting control region (the IG-DMR) and presumably other eutherian specific regulators. In contrast, eutherian zone 6, located between *Gtl2* and *Rtl1*, is smaller than in marsupials, platypus and chicken, implying either that it contracted or that it is resistant to expansion. This latter explanation is favoured, because in marsupials expansion is predominantly due to LINE1s, and in platypus to LINE2 repeats and SINEs. This exclusion suggests an important previously unrecognized eutherian specific function for that zone (Figure 4).

The eutherian specific expansion of zone 7, as for zone 3, is not associated with the insertion of repetitive sequences, compared with marsupials. Rather, zone 7 represents the region located between *Rtl1* and *Mirg*, which, in eutherians, contains approximately 50 miRNA genes and three clusters of C/D snoRNA genes, all expressed from the maternally inherited chromosome [23]. With one exception (see below), our analysis failed to find homologous sequences in marsupials, platypus, or chicken. Instead, the zone contains LINE1 repeats in marsupials and as before, LINE2s and SINEs in platypus (Figure S4). Therefore eutherians acquired transcribed non–protein coding RNAs in a zone that appears resistant to expansion by LINEs and SINEs. Interestingly, the acquisition of snoRNA genes in the imprinted Prader Willi-Angelman syndrome locus also corresponds to the acquisition of imprinting [12].

**Figure 5:**
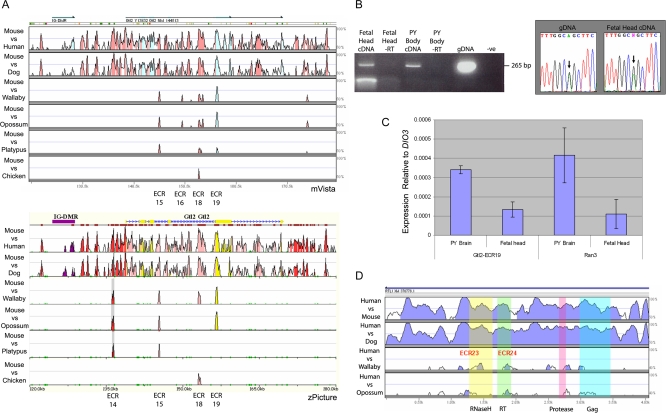
Assessment of Noncoding RNA Transcription (A) Identification of ECRs in the *Gtl2* region in noneutherians. mLAGAN and zPicture alignments of mouse *Gtl2* with human, dog, wallaby, opossum, platypus, and chicken are shown. Four intronic ECRs are identified and one (ECR19) aligns within exon 5 of NM_144513. ECR14 is inverted in the eutherians and was only identified using the zPicture alignment. Weak expression was identified for ECRs14, 15, 18 and 19. RT-PCR for ECR19 in fetal head and pouch young body is shown. (B) Weak expression from ECR19 in tammar wallaby fetal head and pouch young. An A/G SNP was identified in ECR19 and biallelic expression was observed. (C) The expression ratio of ECR19 and Random Primer set 3 relative to *DIO3* in fetal head and pouch young brain as calculated by quantative RT-PCR. (D) A region orthologous to the retrotransposon-derived gene *Rtl1* was identified in marsupials. The mLAGAN algorithm was used to align human *RTL1* with mouse, dog, wallaby, and opossum. Regions with homology of >55% over 80 bp are shown in blue. Regions of human *RTL1* with homology to the Sushi-ichi domains are highlighted. Homology between the eutherian and marsupial regions indicates that *RTL1* inserted into the region before the divergence of eutherians and metatherians.

In the mouse, all the imprinted non–protein coding transcripts in the domain require the imprinting control element and sequences 5′ to *Gtl2* for their activity on the maternally inherited chromosome. They are all expressed in the same orientation, and data suggest that they are at least in part associated with a single long transcription unit [17,24]. ECRs specifically associated with *Gtl2* were identified by phylogenetic footprinting (Figure 5A). Two approaches were undertaken to determine whether *GTL2* and other non–protein coding transcripts were present within the domain; expression analysis of *DLK1-DIO3* intergenic ECRs and the amplification from cDNA of randomly selected sequences from the wallaby region (Figure S5A and Table S3). Five mammalian ECRs were found in the vicinity of *Gtl2*, of which three were common to all vertebrates; one corresponds to exon 5 of NM_144513 (ECR19), and the remainder appear to be intronic. One of the intronic ECRs (ECR18) was previously identified in intron 8 of Y13832 [22]. An additional ECR (ECR14) located close to exon 1 was identified and found to be inverted in eutherians (Figure 5A). This and the three vertebrate ECRs were expressed at very low levels in wallaby tissues, with no transcriptional activity from the other two. RT-PCR analysis of 29 additional, randomly selected sequences in wallaby located between *Gtl2* and *Mirg* identified weak transcriptional activity from five sequences, including one mammalian *Mirg-*specific ECR (Figure S5). Quantitative RT-PCR comparing the relative expression of ECR19 and one of the random sequences (Ran3) with *DIO3* expression in the same samples confirms expression from the *GTL2*-like locus in marsupials is between 1.1 × 10^−4^ and 4.2 × 10^−4^ lower in fetal head and pouch young brain (Figure 5). Polymorphisms located in ECR19 and the *MIRG*-ECR were used to demonsrate that this low level of transcription is biallelic (Figure 5B and Figure S4).

**Figure 6:**
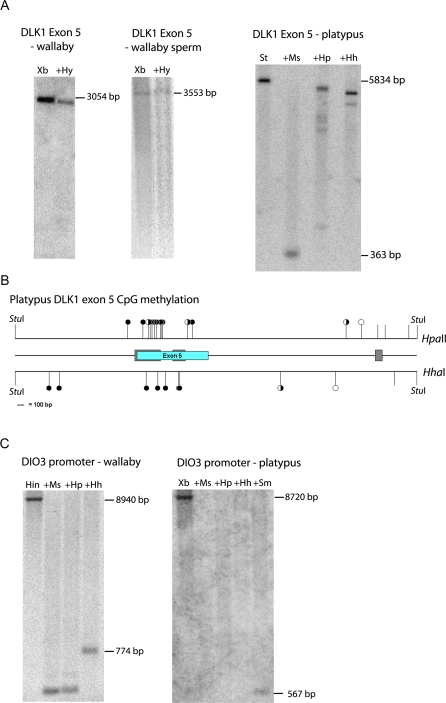
Methylation Analysis of *DLK1* Exon 5 and *DIO3* Promoter in Wallaby and Platypus (A) Hypermethylation was observed in both the wallaby and platypus *DLK1* exon 5 regions. Wallaby genomic DNA from d23 RPY fetus (gDNA) and wallaby sperm gDNA was digested with *Xba*I (Xb), further digested with *Hpy*CH4IV (Hy) and analysed by Southern blot hybridisation using *MeDLK1*Ex5 as a probe. Platypus gDNA was digested with *Stu*I (St) and further with *Msp*I (Ms), *Hpa*II (Hp), and *Hha*I (Hh) and analysed by Southern blot hybridisation using *OaDLK1*Ex5 as a probe. (B) A map depicting the *Hpa*II and *Hha*I sites and methylation status in *Dlk1* exon 5. Black circles indicate methylated sites, white circles unmethylated sites, and half black circles indicate partial methylation. CpG islands in the region are shown as grey boxes. (C) The *Dio3* promoter region is unmethylated in both wallaby and platypus. Wallaby fetal head gDNA was digested with *Hin*dIII and further with *Msp*I (Ms), *Hpa*II (Hp), and *Hha*I (Hh) and hydridised with *MeDIO3*CpG. The methylation-sensitive *Hpa*II and *Hha*I tracks exhibited full digestion indicating the region is unmethylated. Platypus gDNA was digested with *Xba*I (Xb) then with *Msp*I (Ms), *Hpa*II (Hp), *Hha*I (Hh), or *Sma*I (Sm) and hydridised with *OaDIO3*CpG. High CG content results in many *Hpa*II and *Hha*I fragments, which are unmethylated and too small to be resolved on this filter. The smallest *Sma*I site expected was identified, showing the platypus *Dio3* promoter is unmethylated. Control hybridisation with a *OaDIO3* promoter proximal probe identified a fully methylated XbaI fragment of >3 kb in the HpaII, HhaI, and SmaI tracks, confirming the integrity of the genomic DNA in these tracks (unpublished data).

It was of particular interest to determine whether the protein-coding, retrotransposon-like gene *Rtl1* (also known as *Peg11/Mart1*) was present in non-eutherian mammals. *Rtl1* is a member of the Ty3-Gypsy family of LTR retrotransposons with closest similarity to the Sushi-ichi class [25]. In mouse and human, it has lost its LTRs, encodes a protein essential for normal placental development and fetal growth and viability (M. Ito, A. Ferguson-Smith, unpublished data, and [26]), and is expressed from the paternally inherited chromosome. Its levels are regulated by miRNAs processed from an antisense transcript on the maternally inherited chromosome that are 100% complementary to the *Rtl1* mRNA (Figure 1A) [17,27,28]. Another member of this family, *Peg10* located on mouse Chromosome 6, was recently shown to be imprinted in wallaby fetus and placenta (but is absent in the platypus), and its repression on the maternally inherited chromosome is associated with differential methylation in the body of the gene [9]. We could not demonstrate *RTL1* sequences in the platypus or chicken domain. However, we did find sequences related to *Rtl1* in the appropriate position in marsupials but, interestingly, it is extensively degraded with very few regions of homology remaining (Figure 5D). No expression of the most highly conserved region was found in fetal and pouch young tissues (Figure 6A). This suggests that *Rtl1* retrotransposed into the locus prior to the divergence of marsupial and eutherian mammals and, in the absence of functional selection, it degraded in marsupials but acquired a growth regulatory function in eutherians coincident with the evolution of imprinting.

A number of miRNAs that are antisense to *Rtl1* are transcribed from the maternal chromosome in eutherians. Using the miRNA prediction programme miR-abela [29], no miRNAs were found to be conserved between all vertebrates, and none were conserved between eutherians and marsupials. A single predicted miRNA was conserved between the marsupials (74% identity) (Figure S6B and S6C). Interestingly, this was located in the vicinity of the eutherian miR127, which is transcribed antisense to *Rtl1* and along with seven others, contributes to the stability of the *Rtl1* mRNA through an RNAi-dependent mechanism [27]; a function that would not be evident in marsupials that lack this gene. The sequence of the predicted processed miRNA from marsupial miR127 though common to both marsupials is less similar to eutherians and RT-PCR analysis failed to amplify the primary transcript or predicted hairpin from wallaby fetal head or pouch young brain cDNAs (Figure S6D). These data suggest that this is not a functional miRNA, and sequence similarity is due to miR127 being located within *RTL1*.

A small number of conserved CpG islands and CpG-rich regions were found to be shared between eutherians, marsupials, and platypus and their methylation status was determined. They included the promoters of *Dlk1* and *Dio3* and the differentially methylated region in the last exon of eutherian *Dlk1*, known as the Dlk-DMR [16,30]. Each region was analysed by methylation-sensitive Southern blots with genomic DNA from platypus and wallaby and from wallaby sperm. Results are shown in Figure 6. The ECR at intron 5 in *Gtl2* (Y13832) is CpG-rich, and this too was analysed. As in eutherians, the *DLK1* and *DIO3* CpG-island promoters are completely unmethylated on both parental chromosomes. The *Gtl2* ECR is partially methylated on both parental chromosomes in mouse, and has the same pattern in platypus and wallaby. In mouse, the Dlk-DMR is hypermethylated on the paternally inherited chromosome and in sperm, and hypomethylated on the maternally inherited chromosome [16,30]. Platypus and wallaby genomic DNA showed hypermethylation of the locus similar to that seen on the paternal chromosome in the mouse. Wallaby sperm was also hypermethylated. This suggests that the methylation state of the mouse paternal chromosome resembles the methylation state of the mammalian domain prior to the emergence of imprinting and implies that hypomethylation of the maternal chromosome evolved with imprinting.

## Discussion

In eutherians, *Dlk1* and *Dio3* are developmentally important genes that are expressed in numerous embryonic and extraembryonic tissues. Here we have shown that *DLK1* and *DIO3* are both biallelically expressed in marsupial fetus, placenta, and neonatal pouch young. *DLK1* was recently shown to be expressed biallelically in adult brain, liver, and kidney in the South American marsupial, Monodelphis domestica; however, analysis of imprinting in embryonic and extraembryonic tissues was not conducted in that study [31]*.* We also demonstrate biallelic expression of both genes in platypus. Because fetal material is not available, biallelic expression of these genes during platypus development can only be inferred. Together, our results indicate that imprinting of the whole *DLK1-DIO3* domain evolved after the divergence of metatherian and eutherian mammals.

Comparative sequence analysis of the *DLK1-DIO3* region in seven different amniote vertebrates (representing Eutheria, Metatheria, Prototheria, and Aves) demonstrates that the overall genomic landscape in this region is GC-rich in eutherians but not in the other species studied. It has previously been postulated that GC-rich isochores in eutherians were once located on GC-rich microchromosomes in the ancestral amniote [32]. The elevated GC content in eutherians but not in the noneutherian species suggests that the increase occurred in eutherians rather than existing as an ancient isochore.

A number of results suggest that the *DLK1-DIO3* is a recombination hot spot and under purifying selection in eutherian species where it is imprinted. First, elevated GC content correlates with increased levels of recombination [32]. Second, the introns of *DLK1* are shorter in the eutherians than in the noneutherian species (Figure S2B), and decreased intron length is associated with high recombination rates [33]. Third, the reduced SINE content in the eutherian indicates the region is under purifying selection, especially because SINEs are usually associated with GC-rich regions. Interestingly, the region between vertebrate ECR1 and ECR8, which encompasses *Dlk1*, *Gtl2*, *Rtl1*, snoRNAs, and miRNAs, is particularly devoid of LINEs, indicating that this region is under even greater constraint (Table 1 and Figure 4A). Finally, the eutherian *DLK1-DIO3* regions are also all located close to the telomeres, whereas in noneutherian species, they are located mid-chromosome [19]. A correlation of elevated recombination levels at sub-telomeric regions has previously been reported [34–36]; however, it is possible that this sub-telomeric position is the result of increased breakage in GC-rich regions [37]. Imprinted domains have previously been shown to be associated with elevated GC content [38–41], short introns [42], and reduced SINE content when compared to nonimprinted regions in eutherians [21,43]. Our finding that this comparison can be extended to the same domain between mammals that imprint and those that do not strongly suggests that imprinted domains are under purifying selection perhaps to constrain domain size such that *cis*-acting elements can function correctly.

None of the ECRs maps to the position of the eutherian imprinting control element. Whether any of the ECRs plays a functional role in the regulation of the domain is currently under investigation. Those specific to subgroups such as oviparous vertebrates, or the sixteen ECRs specific to therian mammals, might relate to the regulation of specific functions such as the development of extraembryonic structures in therians.

Expression analysis has provided evidence that *Gtl2* and other noncoding transcripts existed throughout amniote evolution, suggesting that *Gtl2* did not arise from an eutherian-specific retrotransposition event that triggered imprinting at the domain as has been previously suggested [31]. Our results show that weak regional non–protein coding transcriptional activity can occur in some places across the domain in noneutherian mammals and suggest that the process repressing the protein-coding genes on the maternal chromosome in eutherians (driven by the imprinting control region upstream from *Gtl2*) facilitated stronger expression from these non–protein coding transcripts. The appearance of functional miRNAs and C/D snoRNAs within the locus may therefore have been a consequence of the acquisition of imprinting with the strongly expressed *Gtl2* gene, providing an ideal host transcript. It is not known whether the duplications that gave rise to the miRNA clusters occurred before or after evolution of imprinting at the locus. Interestingly, a role for these miRNAs in the *trans*-regulation of neural and placental processes has been inferred [44]. A functional role for these transcripts in the regulation of the neighbouring imprinted protein-coding genes also cannot be ruled out. Furthermore, the emergence of a regulatory relationship between *RTL1* and its reciprocally imprinted miRNA-containing antisense transcript is also intriguing. In contrast to the more distal miRNA clusters to which they are not related, these seven *anti-RTL1* miRNAs are not likely to have arisen through duplication/divergence events. Rather, these may have evolved as a host defence mechanism associated with the retrotransposon properties of *RTL1,* and evolved with it to modulate its expression [27] as it acquired an endogenous function.

**Figure 7:**
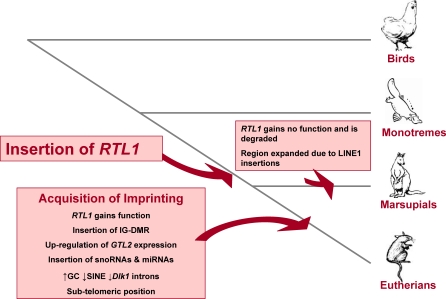
Evolution of the *Dlk1-Dio3* Domain in Mammals. Schematic illustration of the evolution of the *Dlk1-Dio3* domain in mammals. *RTL1* retrotransposed into the region before the divergence of the eutherians and metatherians. In the marsupial lineage, *RTL1* did not gain a function (or lose it) and became degraded. The region expanded approximately 2-fold in the marsupials; this expansion is mainly due to the accumulation of LINE1 repeats. The snoRNA and miRNA clusters arose after eutherian diverged from marsupials but before the mammalian radiation which took place around 98 million years ago. The eutherian region has also evolved many genomic features associated with imprinted clusters. The entire domain has become increasingly GC-rich, whereas a decline in GC content is the general trend in eutherian genomes. There are fewer SINEs than expected in the region, and the introns of the *DLK1* transcript have become shorter. Finally the region has a sub-telomeric position within the eutherian genome whereas in monotremes and marsupials it is in the middle of the chromosome arm. Not drawn to scale

During the course of evolution, the genomic landscape of the *Dlk1-Dio3* region has undergone a number of changes (Figure 7). Most significantly, the region has become imprinted. This analysis has proven that imprinting in this domain emerged after the divergence of marsupials and eutherian mammals. This provides evidence that mammalian imprinting evolved at different loci at different times in response to selective pressures acting on different domains, suggesting an adaptive process. Prior to the divergence of metatherians from eutherians, the Sushi-ichi retrotransposon *Rtl1*, inserted between *DLK1* and *DIO3*, gained no function and was degraded in marsupials. In marsupials, the region expanded 2-fold through the insertion of LINE repeats. As the eutherian lineage evolved through selective regional changes, *Rtl1* evolved into a new gene acquiring a vital function in growth and development. This gain of function may indeed have driven imprinting at the domain, conferred through the acquisition of the imprinting control element. *Gtl2* and associated transcripts became up-regulated on the maternal chromosome in eutherians, and miRNAs and C/D snoRNAs specifically evolved in the region. Once imprinted, gene expression was fixed in the region it underwent purifying selection, correlating with an increase in GC content, reduction in *Dlk1* intron size, and selection against SINE and LINE insertions. Comparison of these results with similar detailed analyses on domains acquiring imprinting prior to the divergence of marsupials and eutherians will provide further insight into the relationships between dynamic changes in genomic landscape and the evolution of imprinting.

## Materials and Methods

### Expression analysis.

RNA was extracted using the GenElute mammalian total RNA miniprep kit (Sigma) following the manufacturer's protocol. cDNA was synthesized using Superscript III RNase H^−^ Reverse Transcriptase (Invitrogen) following the manufacturer's instructions. The RT-PCRs were primed using random hexamer primers or the following gene-specific primers; platypus *DLK1* 5′-GAACGTTTATTTTACAAAAGATAGCTG-3′, wallaby *DIO3* 5′-CGGGCACTCACAGAGTTACA-3′, and platypus *DIO3* 5′-GACTCCGTCTCCGAGAACAT-3′, and 5′-TGAACATCTTACAAAAACCAACAAA-3′. cDNA was amplified using Hot Start KOD polymerase (Novagen), PCR conditions are as described in [19]. For particularly GC-rich regions (e.g., platypus *DIO3*) 1× Polymate (Bioline) was also added to the PCR reaction. Primer sequences and annealing temperatures can be found in Table S3). PCR fragments were gel purified using Qiaquick Gel Extraction Kit (Qiagen) and sequencing was performed.

For ECR and random sequence expression analysis, cDNA was generated as above using random hexamers and PCR amplification performed using either Hot Start KOD polymerase (Novagen) or Taq polymerase (Bioline), using conditions described in [19]. The primer sequences and annealing temperatures can be found in Table S4.

### Allelic discrimination quantitative RT- PCR.

Custom TaqMan assays were produced using the Assays-by-Design facility at Applied Biosystems. 1 μl of cDNA was amplified in a 12.5-μl reaction 1× TaqMan Universal PCR Master Mix (Applied Biosystems) and 1× specific assay as per the manufacturer's instructions. C_T_ (threshold cycle) values for both the VIC and FAM probes were recorded and the difference between them (ΔC_T_) was calculated. Samples were analysed in triplicate. Genomic DNA from homozygous individuals, was used as controls to ensure no cross hybridisation occurred between the two probes. The ΔC_T_ of cDNAs was compared with a standard curve of ΔC_T_ values from two homozygous gDNAs mixed at different ratios (49:1, 9:1, 4:1, 7:3, 3:2, 1:1, 2:3, 3:7, 1:4, 9:1, and 1:49), and the percentage expression from each allele was extrapolated. This method was adapted from [45]. The primers used were as follows: MeDIO3UTR-F, 5′CTTCCCTCCTCCCCAAATTCC-3′; MeDIO3UTR-R, 5′-TGCAGTCAACAAAGTGGAGGAA-3′; + allele probe, 5′-(VIC)-TTCTCTCCTTGGTTTTT-(MGB)-3′; and – allele probe, 5′-(FAM)-TTTTTTCTCTCGGTTTTT-(MGB)-3′.

### SYBR green qRT-PCR assays.

Assays were performed using the SensiMix NoRef kit (Quantace). The amplification of each primer pair was determined using a serial dilution of cDNA (1, 1/5, 1/25, 1/125, 1/625). Reactions were performed in triplicate, and the average C_T_ value of each dilution was used to generate a standard curve. The slope of the curve when plotted to log_10_ was used to determine the efficiency of amplification (E) for each primer set using the following equation: E = 10^(−1/slope)^ and the relative fold expression calculation (2^– ΔCT^) was corrected for the amplification efficiency [46]. The relative gene expression was calculated using the following equation: Expression Ratio = E^ΔCT(sample)^/E^ΔCT(reference)^. Sample reactions were performed in triplicate. Three fetal head samples were used (from between d22 and d25 RPY) and three PY brain samples (from between D17 and D20 post partum).

The primers used were as follows: MeDIO3QF, 5′- CCGAGGGCTACAAGATCTCA-3′; MeDIO3QR, 5′- CACGTTTGTTTGGGGTTCTT-3′; MeECR19F, 5′-GCGGCTTCACAAATTTATTTTC-3′; MeECR19R, 5′-CAACTCTGCACAGATGGATGA-3′; MeRan3F, 5′-CAGCTGGATCCAATTTGACA-3′; and MeRan3R, 5′-TTGGACCATGATCCTGGAAT-3′.

### Methylation analysis.

Genomic DNA was extracted using standard protocols [47]. 10 μg of restriction enzyme–digested DNA was separated on 0.5× TBE 1% agarose gels and transferred to Hybond-N+ (GE Healthcare) nylon membranes. Filters were pre-hybridised in ULTRAhyb solution (Ambion) at 42 °C for a minimum of 2 h. Probes were labelled with [α-^32^P]-dCTP using the Megaprime DNA labelling system (GE healthcare) and added to the hybridization buffer. Filters were incubated at 42 °C overnight, washed to the stringency of 2XSSC/0.1%SDS, and exposed to a phosphoimager screen. Probes used for the analysis can be found in Table S3.

### Sequence analysis.

With the exception of novel platypus and tammar wallaby sequence generated here, sequences for computational analysis were downloaded from the UCSC genome browser [48]. These sequences are: human sequence, build March 2006, chr14:100210000–101150000; mouse sequence, build February 2006, chr12:109850000–110780000; dog sequence, build May 2005, chr8:71946000–72800000; opossum sequence, build January 2006, chr1:315760000–317570000 (reverse complement); and chicken sequence, build May 2006, chr5: 51365000–51832000.

The repeat content for the *Dlk1-Dio3* region in each species was determined using RepeatMasker version: open-3.1.8 [49]. The specific repeat library was used for each species and the default parameters. For the platypus sequence, a command line version of the software was used with a pre-release of the latest library for this species (kindly provided by R. Hubley, RepeatMasker, Institute for Systems Biology, USA).

CpG islands were predicted by CpGplot [50] using the default parameters and a window of 200.

### Evolutionary conserved region prediction.

Two different programs were used to predict ECRs: zPicture [50] and mVista [51,52]. The LAGAN algorithm [53] was used in the mVista alignment. The translated anchoring option (where one or more of the alignments steps are performed on translated sequence) was used, because it can improve the alignment of distant homologues. The default setting of >70% identity and >100 bp in length was used between marsupials and platypus. Greater than 55% identity and >80 bp was used for the alignments of eutherians to other species and chicken to other species. For both programs, human, mouse, dog, and chicken sequences were repeat masked using the species-specific setting. Wallaby, opossum, and platypus sequences were first repeat masked with repeats changed to lower case (softmasking).

Each sequence was used in turn as the base sequence for aligning the others to and the coordinates for the same ECR in each species were identified. The coordinates were recorded for the largest region showing homology between the pairwise alignments of any species. Using this approach, the ECR data were merged between the different pairwise alignments generated by each program and between the two programs. In addition, ECRs that were less than 200 bp apart in all species were merged.

Putative miRNAs were identified in using the miR-abela program [29]. Putative CTCF binding sites were identified using the program FUZZNUC to search the forward and reverse sequence of the *DLK1-DIO3* region in each species. Three motifs were used CCGCNNGGNGNC [54], CCGCGNGGNGGCAG [55], and CCDSNAGRKGGHDS (which is based on the binding motif identified in a large scale analysis of CTCF binding site in human [56]).

## Supporting Information

Figure S1 Overlapping BAC Clones Identified for Sequencing. (A) Complete coverage of the *Dlk1-Dio3* region in Macropus eugenii was achieved through sequencing of BAC clones indicated in red. The thirteen sequenced clones span 1,674,705 bp. BAC details are summarised in Table S1A.
(B) Complete coverage of the *Dlk1-Dio3* region in Ornithorhyncus anatinus was achieved through sequencing BAC clones indicated in red. Seven clones spanning 795,237 bp were sequenced. BAC details are summarised in Table S1B.
The Macropus eugenii genomic BAC library was from the Arizona Genomics Institute (average insert size 166 kb) covering 11.36 genome equivalents and cloned into the *Hin*dIII site of pCUGIBAC1. The Ornithorhyncus anatinus BAC library was from Clemson University Genomics Institute (average insert size 143 kb) covering 11 genome equivalents. It was constructed in the HindIII site of pCUGIBAC1. Libraries were screened with probes for *Dlk1* and *Dio3*. A *Dlk1* probe was generated for wallaby by searching the Monodelphis domestica trace archive with human *DLK1* with a probe designed against the most conserved sequence. *DLK1* sequence for platypus was identified by searching the platypus trace archive with chicken *Dlk1* sequence and a probe was generated to this platypus sequence*. Dio3* probes amplified from wallaby and platypus were generated after identification of conserved primers from alignments of human, mouse, rat, and chicken *Dio3*. BACs identified from library screening were amplified to confirm presence of probe sequences, and HindII digested and fingerprinted allowing them to be aligned and assembled into a contig. One BAC from each end was selected for shotgun sequencing. Using end sequence or fully sequenced BACs, new probes were generated and the process repeated until complete coverage was achieved.
(1.6 MB TIF)

Figure S2 Exon Sequence, Structure, and Polymorphism for Wallaby and Platypus *DLK1*
(A) mRNA sequence of wallaby and platypus *DLK.* The protein coding region (CDS) is highlighted turquoise and is in uppercase. The 3′ UTR is highlighted in yellow. The full extent of the 3′ UTR was not established and is illustrated up to a predicted poly adenylation signal (green). All of the splice sites have an intronic GT in the donor site and intronic AG in the acceptor sites (red). The polymorphisms identified within the genes are in bold and surrounded by square parentheses.
(B) Schematic alignment of the intron-exon structure of the gene in human, mouse, wallaby, opossum, platypus, and chicken indicates that the two 3′ marsupial introns are greatly expanded compared to those of the other vertebrates. In eutherians, the *Dlk1* gene span is less than 1% of the entire domain (from the start of *Dlk1* to the stop codon of *Dio3*). However, in the noneutherian species analysed, the *Dlk1* gene span is greater than 2% of the region.
(C) Sequence traces of genomic DNA and cDNA from heterozygous individuals confirm that the gene is biallelically expressed in wallaby and platypus. Sites of single nucleotide polymorphism are indicated with red arrows.
(25.5 MB TIF)

Figure S3 Wallaby and Platypus *DIO3*
*DIO3* in all vertebrates contains a single exon. The sequence of the *DIO3* gene in wallaby and platypus is shown. The coding sequence is highlighted turquoise and is in uppercase. The 3′ UTR is highlighted in yellow. The full extent of the 3′ UTR is illustrated up to a predicted poly adenylation signal (green). The polymorphisms identified within the genes are in bold and surrounded by square parentheses.
(2.62 MB PDF)

Figure S4 Comparative Repeat Content of Regions Showing Expansions/Contractions between Therians Inter-zone repeat content of regions which show expansions and lack of expansion (contractions) in eutherian and marsupial mammals. LINE elements are subdivided into four families (L1, L2, L3, and RTE) to illustrate the different contributions made to overall LINE content.
(739 KB TIF)

Figure S5 Assessment of Noncoding RNA Transcription in Wallaby (A) Schematic representation of the *Dlk1-Dio3* domain indicating locations of ECRs and randomly selected loci assessed for expression. Position of the primer pairs used for the RT-PCR analysis areindicated by black bars below the horizontal line. The positions of primer used to analyse ECR expression are shown in red. Above the horizontal line are ECRs. Turquoise, ECRs conserved in all vertebrates studied; pink, ECRs conserved in all non-eutherians studied; blue, other ECRs. The extent of the expression is indicated by a broken green line.
(B) RT-PCR with primers designed to ECR36 (located within an intron of *Mirg* in mouse). Expression was seen from wallaby fetal head.
(C) A G/T SNP was identified in one wallaby PY sample. Sequence trace data demonstrate biallelic expression of ECR36.
(3.99 MB TIF)

Figure S6 Wallaby Lacks miRNAs but Has Remnants of *RTL1*
(A) RTL1 is not expressed in the wallaby. Primers, designed to the reverse transcriptase orthologous region which is expressed in eutherians, failed to amplify cDNA from wallaby placenta, fetal head, or pouch young brain. BYS, bilaminar yolk sac; TYS, trilaminar yolk sac; PY, pouch young.
(B) Sequence alignment of human and mouse miR-127 with the tammar wallaby and opossum miRNAs as predicted bythe miR-abela program [29].
(C) Comparative secondary structure; the known mature miRNA regions are indicated by red parentheses. Orthologous regions to mature miRNAs are indicated by blue parentheses
(D) The pre-RNA region was not amplified from wallaby pouch young brain cDNAs.
(4.95 MB TIF)

Table S1 Wallaby and Platypus Sequenced Clones (A) List of sequenced wallaby BACs including accession numbers and finished lengths. (B) List of sequenced platypus BACs including accession numbers and finished lengths.
(15 KB XLS)

Table S2 The ECRs Identified in the *Dlk1-Dio3* Region in Human, Mouse, Dog, Wallaby, Opossum, Platypus, and Chicken Each ECR is give a number from 1 to 141. In addition, the 31 vertebrate ECRs are indicated by V1 to V31. The coordinates of each ECR are given for their position in the analysed sequences. ECRs, which are inverted in at least one species are indicated by (–). One ECR (ECR64) is duplicated in the human sequence.
(66 KB XLS)

Table S3 Summary Expression Analysis Indicating Absence or Presence of Expression (17 KB XLS)

Table S4 List of PCR Primers Used for Expression Analysis and Southern Hybridisation Probes The PCR conditions used are indicated.
(26 KB XLS)

Table S5 List of Wallaby Samples Used in This Analysis By manipulating the reproductive cycle of the tammar wallaby, it is possible to recover animals at specific stages of development. This is because lactating mothers have a second fertilised embryo arrested in diapause. During the wallaby breeding season, pregnancy can be initiated by the removal of the pouch young (RPY), which reactivates the blastocyst from diapause. Pregnancies are dated relative to the day of pouch young removal and are prefixed with d (e.g., d23). Pouch young are dated from days post parturition and are prefixed by a D (e.g., D10).
(14 KB XLS)

## Abbreviations

ECR: evolutionary conserved region
IG-DMR: intergenic differentially methylated region
LTR: long terminal repeat
miRNA: microRNA
snoRNA: small nucleolar RNA
UTR: untranslated region
