# Supplementary material for: The Evolution of the DLK1-DIO3 Imprinted Domain in Mammals
Source: PLoS Biol. 2008 Jun 3;6(6):e135. doi: 10.1371/journal.pbio.0060135 (PMC2408620; doi:10.1371/journal.pbio.0060135)
Supplement: Supplementary file 1 [file pbio.0060135.sg003.pdf]

Wallaby *DIO3*

tataaatccagtttaaggggaagcgggatctcagATGAGCAGCCCGGACCCAAGTCGGCTAGGGGA
GCGAGGGGGTGGGGCCCAGGGCGGCCGCGGCCGCAGCTGCCC**[G/A]**GCTGCGCCGCGGCTAGCCG
GCAGGCTCCACTCCCTCCTGGTTCACACCTTGGGCTTTCTTACCCAGGTGGCCGCCTGCTGTTTCC
TGGGTCCGAGATTCCTGGCCACAGCGGTGACGCTGTGGCTACTGGACTTCCTGTGCATCCGCAAGA
AGGTGCTGATGAGACACCGGCGCGAAGGCGACGCTGAGGACCCCTGGAAGGCGGGTGGGGGCGGGG
GCGGGGGTGGAAGAGAGGAGGAGGATGCGGCAGCTGTTATCTTGTCCCCGGGGCTACCCCCCGATG
ACCCGCCAATCTGGGTCTCTGACTCCAATCGGTTCTGCACTATAGAGTCGCTGAAGGCTGTCTGGC
ACGGGCAAAAGATGGACTTCTTCAAAACTGCCCACAAGGGCTCCCCAGCCCCCAATCCCGAGGTCA
TTCAGCTGGATGGACTTAAAAGACTGCGCATCCTAGACTTTGCGCGGGGCAGCCGGCCGCTGGTCC
TCAACTTCGGCAGCTGCACCTGACCCCCGTTCACGGCGCGGCTGCGCGCCTTCCAGCATCTCGCCT
CCACCTTCCTAGACATCGCCGACTTCTTGCTCGTCTACATCGAAGAAGCCCACCCTTCCGACGGCT
GGGTCACCTCAGATGCAGCCTACGACATCCCCAAGCACCAGTGCCTTCAGGACCGGCTGCGCGCCG
CCCGGCTCTTGCAGGAGGGCGCACCGGGATGCTTGCTCGCGGTGGACACCATGGACAACGCGTCCA
GTGCAGCCTACGGTGCCTACTCTGAGCGGCTCTATATCATCCAGGAGGCCCGAGTCATGTACCAGG
GGGGACGAGGCCCCGAGGGCTACAAGATCTCAGAACTGAGACACTGGCTCGACCAGTACAAAGACC
GCCTGCAGAGCCCACCGGCCTCCGTGGTCCTACCTGTGTAAttgaccgtttccaaaacaaagaacc
ccaaacaaacgtgcaattgaaggttttcaatcacagaaagtgacagttacacaaaagaaggggaa
gggagaattagcccatgggggagggggttccagtgggggaaacacttctctcttccctcctcccca
aattcctttttctctc**[ctt]**ggtttttttcctccactttgttgactgcagtgcgaacattgtat
atgttgcgcgtgtgcatgcgtgtgtaaacctgtgtaactctgtgagtgcccgttgtttcccgcaga
gagaagacacccccgccacctgggacaactcaagctctacggggccaatccttgcttgcctgagaa
gaaggaaggaaggaaggaaagaaggagggaaggaaggaaggaaggaaggaaggaaggaaggaagga
aggaaggaaggaaggaaggaaggaaggaaggaaggaaggaaggaaggaaggaaggaaggaaggaag
ggaaaggggggagagagagagagagagagagagaattagagtcctaaataagtgtgaagaattgta
ctgccaagtatcccgatgtgtgactgtaatctctgctcactggttcgctggcctgtgtgtgtgcgc
gcacgcgtgtgatccgatgtctctcggtgtgtgactttttaaaggggagtctgggctgaccctgct
cctcccagcccacttctcttctttgtttctaccaattccgtattgcccagattctttctgtctcgt
aatctcccaaggtgtgtgtatgtatgtttgtgaaagtgtgtatgcgggtgggggaggggggaagag
tccggaagaagaggggacttggcgggggaggagggttgggttgcgcgcgaagtgagtgtctagtta
gctttttctcatcccggttctggcacttctttggtccctctggtagtgacgacctgtttcgaattg
gtcccagaccaggaaaccgtgtctgatattcttagtgacctagaagtgccctaaccgcgagagaaa
aatgagtgtttaatgtatttataaaagtggttgggaaagctaggttgggggtgggggtgggagggc
tctcgggtgggttttgacgatctttgaatctagaactggggcgggagagggagggttcctagttgg
agggctcttggggccgtttgggtgatcttgagcctatctggcttggagaacctagcttggggaata
taacgattttggatcttgagtgtagggttagttatcccctatttgaaggaatacctagttctgatg
tactgtggaaagaaatcatgcaattcatcggtcccttgctcaggggtgaacgagagcaggaatcta
gacctttgatagaaataaa

Platypus *DIO3*

tataaaatgggctcaaggagacgcgggatctcagatgagccggcgggaccctgacgggccgggaag
gagggggggccgggggagccaccgggccgggccgccagctccagccaccccgccgccaccccccgc
gtcgacccccgcgtcgaccgccggtccggcgccATGCTCCACTCGCTCCGCGTTCACACCTCGGCG
CTCCTCGCCCAGGCGGCCGCCTGCTTCCTCCTCTTCCCCCGATTCCTGCTCACCGCACTGATGCTC
TGGCTCCTCGACTTCTTGTGCATCCGGAAGAAAGTCCTGCACGGCCCCGGGGAGGCGGGGCCGGAG
GCGGAGCAGGGCCCGCAGCGGGCCCGGGACGGCGAGGCCCCGGCCGCCGGGCCCGCCCCCCCGGAG
CCGCCCCCCGACGACCCCCCGGTGCTGGTGTCCGACTCCAACCGCCTGTGCACCCTGGAGTCGCTG
AAGGCCGTGTGGCACGGGCAGAAGCTGGACCACTTCAAGTCGGCGCGGGTCGGGGCCCCGGCCCCC
AACCCCGTGGTGGTGGCGCCGGACGGGCCCACGCGGCGTCGCCTCCTGGACTTCGCCCGCGGGGCC
CGGCCCCTGGTCCTCAACTTCGGCAGCTGCACCTGACCGCCGTTCATGGTCCGGCTGCGCTCCTTC
CAGCGGCTGGCCGCCCGCTTCCTGGACGTGGCCGACTGCCTGCTGGTCTACATCGAGGAGGCGCAC
CCGTCCGACGGCTGGGTCAGCTCCGACGCCCCTTACGACATCCCCAAGCACCGGTGCCTGCAGGAC
CGGCTCCGCGCCGCACGGCTGATGCAGCGGGGGGCCCCCGGCTGCCGGCTGGCCGTCGACACCATG
GACAACGCGGCCAGCGCCGCCTACGGCGCCTACTTCGAGCGCCTCTACGTCGTGCAGGACGCCCGG
GTCGTCTACCAGGGAGGCCGCGGGCCCGAGGGCTACAAGATCTCCGAGCTGCGCCTCTGGCTCGAG
CAGTACCGCCTCCGGCTGAGGGGACCCGGCCCCGCCACCGCCGCCGCCGCCGCC**[GCC]**GTCCTCG
ACGTCTAAacccctcccctcccctctctcccccccacccccgacggatcgggccttagggacagtca
gtcctccagcgtcataataaaagatcaccgtcccaatcataagggaaagtgacaataaaagaggag
acagagggacaaaaaaaattcatacatggagaaggtcctgtttaaaaaaaaaaaaaaagcccctcc
cctccagaaaaaaaaaaaatcatcatccttatgactggtattattactttagtggacggccgggtcc
gcctctttctctcttctttccatctctcggctctccatccttcccgccctgtccccggtcccctcc
ccatcccggactctgctcagcgcaggcggctgcggtgtgaggtttttttttgggggggggggagg
ggagggatttttgtgtacgtgcgtgtaacttcccccagcgaggagtgctagtccttccccccctcaa
ccctccgtgtccgcaaatccacgccggtccgccaaacgagctcggttctgaactgcctttccaagg
gtctctgttttctgttttaaaaaaaaaatatatagttgagaagcatcgcggggcgggatggggtcgg
gggggggggggattctcttctgggagtggggtgggggctggggtagtcgaagtggtcgttggcttg
tttgtttgtttgttttcatggaaaatctaggtcgggtcttctgcccaaatgctggagaacccccctc
tccagggtgcgtgttcgtgtgagcacgtgggtgtgcccgtctgtgtgtgtgcgattcgggagaagg
cgctgtggcctcacaggtgtgtatgcgagggtgtctgtgtgtgtgtctgtcgggagaaggcgttgt
aggtacacaggtgtgtatgcgaggggtgtctgtgtgtgtgtctgtcgggagaaggcgttgtaggta
cacaggtgtgtatgcgagggtgtctgtcgggagaaggcgtttgtgggtacacaggtgtgggtgagt
gggtgggtgtatgtgtgtcgggagaagacgttgtgggtacacaggtgtggatgtg**[tgtggggcag**
tgtatatgcgtgtgggtcgggagaaggcgtcgtgggtacacaggtgtggatgtg]cgggtgtgggt
gggtgggtgtgtcggggcccagaaagtcccggacagaagtcgttgctgcgtccgtcgctcgcccac
gggaattggccagaagcctccctcttcgcccgtgacgaccggtctcgaaatggtcccgcaaccagg
agaccgcgtccgatgttctcggagacggagtccgaagccgctcccagggccaggaaccaagcgtcc
tatttatggggtgcggggtgggaggggtacggggtgggctgggg**[g/c]**gggggaggtgtccgttc
ggactgtttcgtttttgttggtttttgtaagatgttcactaatttttctatgaactttttttataa
taaa
